# Hydrogen Sensing with Ni-Doped TiO_2_ Nanotubes

**DOI:** 10.3390/s130708393

**Published:** 2013-07-01

**Authors:** Zhaohui Li, Dongyan Ding, Qiang Liu, Congqin Ning

**Affiliations:** 1 Institute of Microelectronic Materials and Technology, School of Materials Science and Engineering, Shanghai Jiao Tong University, Shanghai 200240, China; E-Mails: lizhaohui@sjtu.edu.cn (Z.L.); lqshjt@sjtu.edu.cn (Q.L.); 2 State Key Laboratory of High Performance Ceramics and Superfine Microstructure, Shanghai Institute of Ceramics, Chinese Academy of Sciences, Shanghai 200050, China; E-Mail: cqning@mail.sic.ac.cn

**Keywords:** TiO_2_, nanotubes, doping, hydrogen sensor

## Abstract

Doping with other elements is one of the efficient ways to modify the physical and chemical properties of TiO_2_ nanomaterials. In the present work, Ni-doped TiO_2_ nanotubes were fabricated through anodic oxidation of NiTi alloy and further annealing treatment. The hydrogen sensing properties of the nanotube sensor were investigated. It was found that the Ni-doped TiO_2_ nanotubes were sensitive to an atmosphere of 1,000 ppm hydrogen, showing a good response at both room temperature and elevated temperatures. A First-Principle simulation revealed that, in comparison with pure anatase TiO_2_ oxide, Ni doping in the TiO_2_ oxide could result in a decreased bandgap. When the oxide sensor adsorbed a certain amount of hydrogen the bandgap increased and the acceptor impurity levels was generated, which resulted in a change of the sensor resistance.

## 1. Introduction

There is an ever-increasing demand for gas sensors in various fields [1,2]. Particular attention has been devoted to the monitoring of hydrogen (H_2_) mainly due to the wide application of hydrogen gas in either a clean energy source or in many chemical plants. As reported in many literatures, metal oxide nanofilms are potential candidates for hydrogen sensors as they played an important role in the last few years as sensing materials for various kinds of gases [3–5]. TiO_2_-based gas sensors have been widely used because of their inert surface properties and the fact their electrical resistance could change after adsorption of hydrogen gas. For example, highly-ordered TiO_2_ nanotubes work as a hydrogen sensor. Hydrogen molecules could be chemisorbed into the grain boundaries and pick up electrons from the conduction band to create a space charge layer among the grains. This will lead to the formation of Schottky barriers at the grain surfaces and thus a decrease of conductivity of the oxide materials [6–8].

However, as a wide bandgap n-type semiconductor material, anatase TiO_2_ (Eg ≈ 3.2 eV) suffers from poor conductivity and this usually causes increased resistance of electronic components when working. Therefore, it is probably hard for anatase TiO_2_ to be considered an ideal semiconducting material for wide use in detecting hydrogen gas. However, it has been demonstrated that element dopants can effectively address this problem. According to previous reports, TiO_2_ doped with metal or non-metal elements usually has a smaller grain size and a larger specific area, which leads to enhanced sensing properties in comparison with undoped TiO_2_[9–12]. The effect of Ni doping in TiO_2_ oxide has been investigated by various groups. Kim [13] and Yao [14] *et al.* investigated the photocatalyst properties of Ni-doped TiO_2_. With Ni-doping the phase composition and the lattice parameters of the TiO_2_ films changed. It resulted in an expanded optical absorption range of the film and a shift to the red with increase of doping degree [14]. Impurity energy levels were generated in the Ni-doped TiO_2_ and bandgap narrowing could be found through using *ab initio* band calculations.

To date, little work has been reported on the hydrogen sensing properties of Ni-doped TiO_2_ oxides. Thus, in the present work, the hydrogen sensing properties of Ni-doped TiO_2_ nanotube arrays fabricated through an anodization and annealing process were investigated. In addition, to establish the influence of Ni doping on the semiconducting properties of TiO_2_, we calculated approximately the energy band of Ni-doped TiO_2_ and the oxide adsorbing certain amounts of hydrogen. The corresponding electronic parameters were investigated to study the hydrogen sensing behaviors. We demonstrate that the Ni-doped nanotubes could have a good performance with high sensitivity and quick response in detecting hydrogen atmospheres at room and higher working temperatures.

## 2. Experimental Section

Equiatomic NiTi (nominal composition: 50.8 at% Ni) plates with a size of 15 mm × 10 mm × 1 mm were first ground and polished with 2000# SiC emery papers, and then ultrasonically cleaned with absolute alcohol. Finally they were rinsed with deionized water and further dried in a N_2_ stream. Electrochemical anodization was carried out with a non-aqueous electrolyte of 5% ethylene glycol/glycerol containing 0.15 M (NH_4_)_2_SO_4_ and 0.2 M NH_4_F. The anodization was conducted for 90 min with an anodization voltage of 20 V. The anodized samples were then annealed at 425 °C for one hour in air to obtain crystallized nanotubes.

Circular Pt electrodes with a thickness of 200 nm were deposited onto surfaces of the crystallized nanotube samples through sputtering. Conductive wires were connected to the Pt electrode with conductive paste. The nanotube samples (with corresponding alloy substrate) were fixed on PCB and two Pt electrodes were connected to the Cu-pads of PCB. The above nanotube sensor was a resistive sensor. A Keithley (OH, US) 2700 multimeter was used to test the resistance variation of the nanotube sensor in atmospheres of N_2_ containing certain concentration hydrogen. All sensing tests were carried out at constant humidity of 45%–50% in air since N_2_ background atmosphere could not support a repeatable sensing response of the oxide sensor. The sensor was put on a hot plate and the testing atmosphere was feed directly toward the sensing surface. The gas tube had an outer diameter of 8 mm and the distance between the tube mouth and the sensor was 10 mm. Total flow rate of the testing atmosphere was 1 L/min.

Surface morphologies of the as-anodized and calcined nanotube samples were examined with a Scanning Electron Microscope (SEM, FEI SIRION 200, OR, US) equipped with Energy Dispersive X-Ray analysis (EDXA, OXFORD INCA, Oxford, UK). Surface compositions and composition distribution along the depth of the Ni-doped nanotubes were characterized with X-ray photoelectron spectroscopy (XPS, ESCALAB 250, MA, US).

Geometry optimization and total energy calculations were done using the CASTEP package [15]. The generalized gradient approximation (GGA) as proposed by Perdew, Burke, and Ernzerhof (PBE) was applied [16], combined with Vanderbilt ultrasoft pseudopotentials [17]. Based on our XPS analysis result doped structure with Ni atoms at anatase TiO_2_ sites were modeled using the 3 × 1 × 1 supercell, in which only one Ti atom was substituted by Ni atom. The maximum plane-wave cutoff energy was taken as 380 eV. Two hydrogen atoms were added to the supercell unit to approximately simulate a sensor detecting a 1,000 ppm hydrogen atmosphere.

## 3. Results and Discussion

### 3.1. Characterization of Oxide Nanostructures

**Figure 1.**
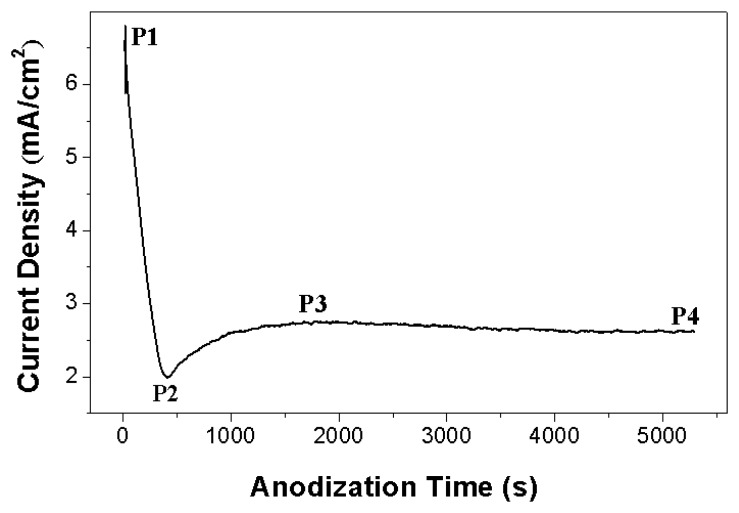
Anodic current density *vs.* anodization time of the anodization process.

Figure 1 shows the anodic current density versus time curves recorded during anodization of NiTi alloy. The anodization current decreased rapidly from 7 mA to 2 mA (point P1 to P2), which corresponded to the formation of a barrier oxide at the alloy surface, leading to a current decay.

At the stage of current increase to a peak value of 2.8 mA (point P2 to P3), the pores of the oxide film grew randomly. Due to the growth of the pore the active area increases and the current increases. After the peak point the current decreased to reach a nearly steady-state value (point P3 to P4), which indicated that the individual pores started to interfere with each other and compete for the available current and then self-assembly oxide nanofilm could grow on the alloy substrate. The steady-state stage corresponds to a process with equal dissolution rate and formation rate of doped TiO_2_ film.

**Figure 2.**
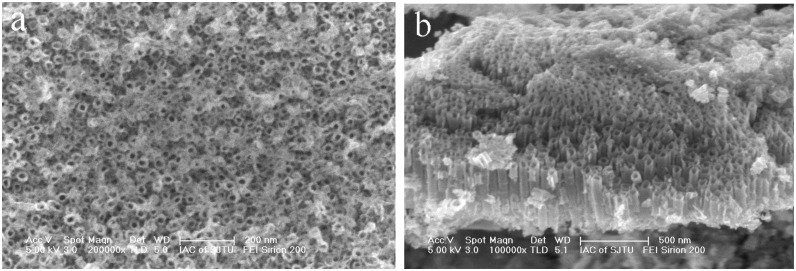
Anodic Ni-doped TiO_2_ nanotubes fabricated at 20 V. (**a**) Surface morphology, (**b**) Cross-section image.

Figure 2 shows the surface morphology and cross-sectional images of the Ni-doped TiO_2_ nanotube arrays prepared through 1.5-h anodization and further heat-treatment at 425 °C in air. It can be found that the nanotubes could still retain their nanotubular structures after the crystallization treatment at 425 °C. And large-area open-ended nanotubes could be observed on top of the NiTi alloy, although the top surface of the Ni-doped TiO_2_ nanotubes was slightly covered with ultrathin oxide films. Average inner diameter of the nanotubes was 65 nm and average length of the nanotubes was around 500 nm.

**Figure 3.**
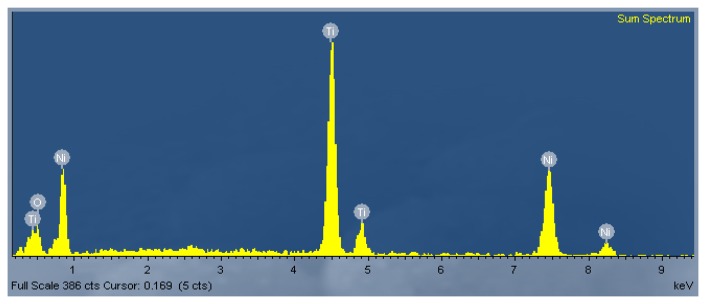
EDX pattern of the Ni-doped TiO_2_ nanotubes.

Our EDX analysis of the as-annealed nanotubes (Figure 3) revealed that the nanotubes consisted of three elements including Ni, Ti, and O. XPS analysis of the chemical composition of the nanotubes further confirmed the formation of Ni-doped TiO_2_ nanotubes on the NiTi alloy susbtrate.

The atomic percentage of the Ni, Ti and O element at the top surface of the Ni-doped TiO_2_ nanotubes were 6.82%, 19.35% and 73.83%, respectively. A depth analysis of the Ni-doped TiO_2_ nanotubes indicated that the atomic percentage of the Ni, Ti and O element were 8.02%, 25.59% and 66.4%, respectively after etching for 25 s. The chemical compositions of the three elements only slightly varied along the longitudinal direction of the nanotube arrays.

**Figure 4.**
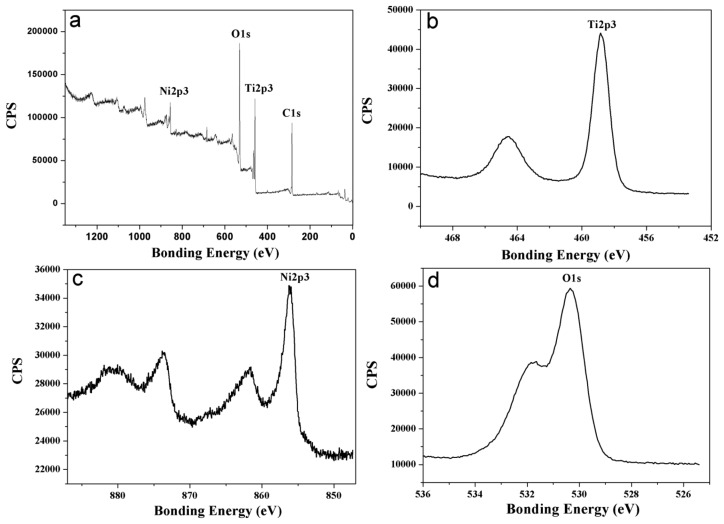
XPS analysis of Ni-doped TiO_2_ nanotubes annealed at 425 °C: (**a**) Survey spectrum, (**b**) Ti2p scan curve, (**c**) Ni2p scan curve, (**d**) O1s scan curve.

Figure 4 presents a survey spectrum and detailed spectra of Ti2p, Ni2p and O1s for the original surface of the Ni-doped TiO_2_ nanotube arrays annealed at 425 °C. According to the Ti2p peak at 458.8 eV and Ni2p peak at 856.14 eV, the Ti element in the oxide nanotubes mainly existed as Ti^4+^ and the Ni element in the oxide nanotubes mainly existed as Ni^5+^. The presence of Ni^5+^ ions was important for the control of free carrier density [14]. Carrier generation in doped TiO_2_ films was generally interpreted to occur from the substitutional Ni^5+^ ions donating extra free electrons [10].

### 3.2. H_2_ Gas-Sensing Properties

**Figure 5.**
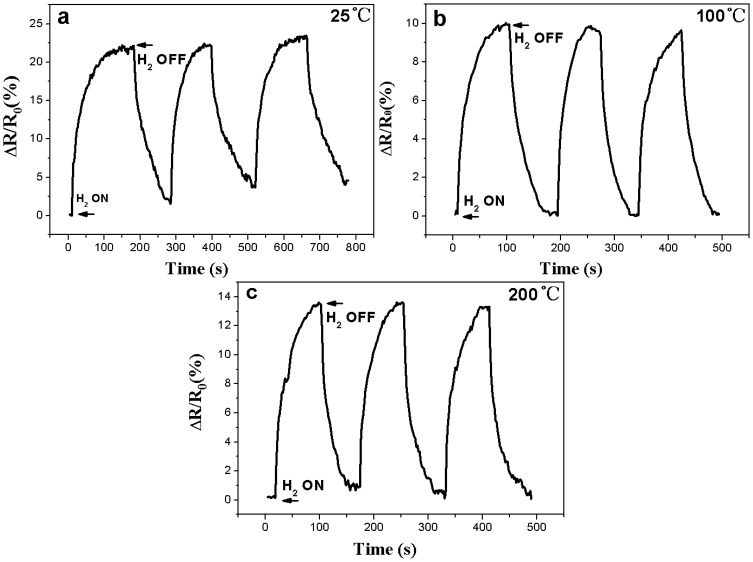
Saturation responses of the oxide nanotubes to a 1,000 ppm hydrogen atmosphere tested at: (**a**) 25 °C, (**b**) 100 °C, (**c**) 200 °C.

Figure 5 shows typical response curves of the nanotube sensor tested with the reducing atmosphere containing 1,000 ppm hydrogen in air. It was found that the nanotube sensor presented a good response working at either room temperature, 100 °C and 200 °C. The resistance of sensor increased rapidly after exposure to hydrogen-containing atmosphere. The response time of the sensor tested at 25 °C was about 170 s. The sensor presented a linear drift of the sensor's resistance. At the relatively lower working temperature of 100 °C, the response time (the time required for the sensor to reach 90% of the saturation value) of the Ni-doped TiO_2_ nanotube was around 100 seconds and a 9.8% change in resistance was found. After the working temperature increased to 200 °C, the response time was still around 80 seconds but the change in resistance increased to 13.7%.

**Figure 6.**
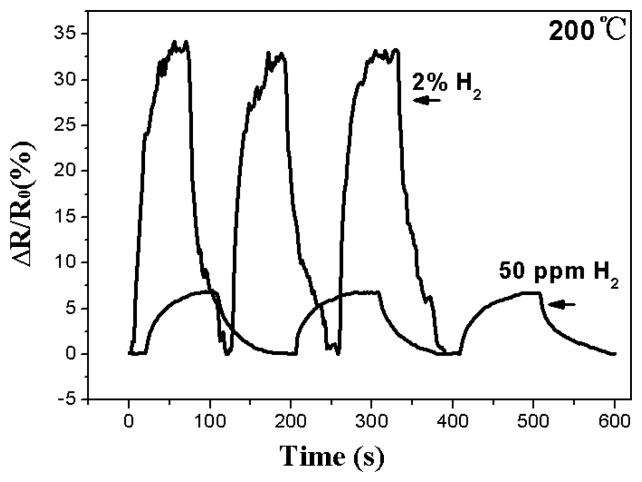
Response curves of the Ni-doped nanotubes for different hydrogen atmospheres.

Figure 6 shows typical response curves of the nanotube sensor tested in either high or dilute concentration hydrogen atmospheres at 200 ° C.

The Ni-doped TiO_2_ sensor demonstrated a good sensitivity for the 50 ppm or 2% hydrogen atmospheres. When the baseline drift phenomena disappeared at elevated temperatures (between 100 and 200 ° C), an increased operating temperature of the nanotube sensor should help to accelerate the diffusivity of the hydrogen atoms into the surface of nanotubes and thus lead to a higher sensitivity [8,18].

The response (ΔR/R_0_) of the Ni-doped TiO_2_ nanotube sensor is defined as follows:

(1) $$\Delta R/R_{0}=(R-R_{0})/R_{0}$$

where R_0_ is the resistance of the sensor before exposing to the hydrogen-containing atmosphere, and R is the resistance after exposing to the hydrogen-containing atmosphere.

In general, anatase TiO_2_ behaved as an n-type semiconductor, with a decrease of resistance in a reducing atmosphere such as H_2_ and CO [2,19,20]. In the present work, the resistance increased when the Ni-doped TiO_2_ nanotubes were exposed to the hydrogen-containing atmosphere and the Ni-doped TiO_2_ nanotubes demonstrated a p-type semiconducting behavior, which was totally different from that of undoped TiO_2_ nanotubes. The effect of Ni-doping on the semiconducting properties of TiO_2_ oxide was similar to those of Cr-doping and Nb-doping oxide [21–23]. For example, Li *et al.* prepared Cr_2_O_2_-TiO_2_ thin films through the sol-gel process. The response of the Cr-doped thin films to O_2_-containing atmosphere revealed the change of conductivity of TiO_2_ from n-type to p-type at high partial pressure of oxygen when the Cr dopant quantity increased [22]. Ruiz *et al.* fabricated Cr-doped TiO_2_ and found that the thin film doped with 10% Cr exhibited a p-type conductivity [24]. In addition, Mowbry *et al.* found that non-metal element dopants like nitrogen could give rise to n-type or p-type doping but boron always leaded to n-type doping in TiO_2_. We believed that Ni dopant should have caused the change of conductivity of TiO_2_ oxide.

### 3.3. First-Principles Calculations

**Figure 7.**
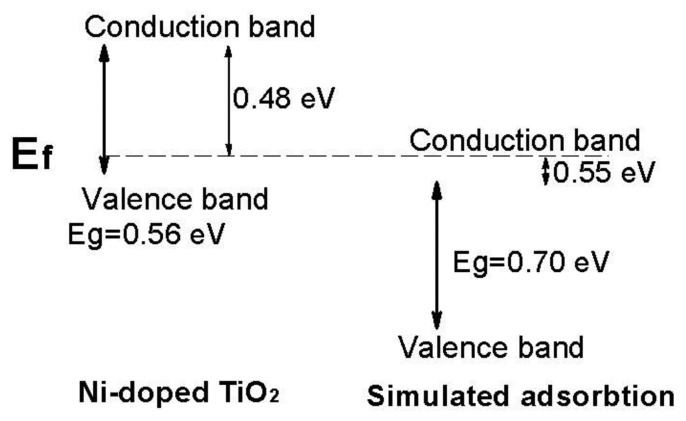
Schematic diagram of energy levels showing the relative position of the Fermi level (E_f_) with respect to the minimum conduction band and maximum valence band with or without hydrogen adsorption. The dotted line corresponds to the Fermi level.

Figure 7 shows the bandgap of the Ni-doped TiO_2_ and simulated sensor in response to 1,000 ppm H_2_. After Ni doping, the bandgap of the as-doped TiO_2_ reduced to 0.56 eV and it was smaller than the theoretical value of pure TiO_2_[25,26], which means that lower bandgap values could improve the electrical conductivity of the sensors.

After the oxide film senor adsorbed a certain amount of hydrogen, the bandgap increased from 0.56 to 0.70 eV. This indicated that, with increase of Eg, electrons will need more energy to excite from valence band to conduction band and simultaneously it showed greater resistance in the conducting characteristics.

**Figure 8.**
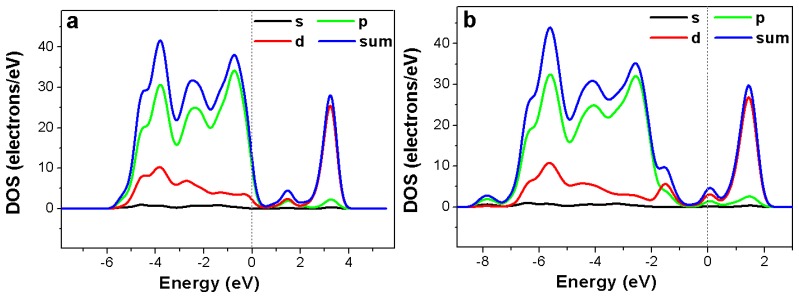
Calculated total and partial density of states of Ni-doped TiO_2_: (**a**) without hydrogen adsorption, (**b**) with hydrogen adsorption.

Figure 8 shows total and partial density of states of Ni-doped TiO_2_ with and without hydrogen adsorption. In the Ni-doped TiO_2_ sensor with or without hydrogen adsorption, the acceptor impurity level could be observed, which means that trapped hole centers were generated. As a result, transition electrons could be captured by the holes and electrical conductivity of the oxide sample could vary. After the doped oxide adsorbed a certain amount of hydrogen the bandgap increased, which resulted in a decrease of electrical conductivity of the system and the increase of sensor resistance. In our experiments, we had found that the sensor resistance increased after hydrogen adsorption. This is in good agreement with the above bandgap simulation results.

The above results revealed that the Ni-doped TiO_2_ nanotubes were sensitive to hydrogen-containing atmospheres at operating temperatures ranging from 25 °C to 200 °C. Generally, the operating temperature of traditional TiO_2_ oxide sensors lies between 200 °C and 500 °C, which often limits the wide application of such kinds of gas sensors. Therefore, the Ni-doping modification of semiconducting TiO_2_ oxides with nanotubular structures demonstrated great advantages of enhancing hydrogen sensing properties, especially the reduction of operating temperatures. Such a good hydrogen sensing performance shown by the Ni-doped nantubes suggests the use various kinds of Ni-doped TiO_2_ nanostructures as hydrogen sensors.

## 4. Conclusions

In summary, Ni-doped nanotubes were fabricated through anodization and annealing at 425 °C. In response to a hydrogen-containing atmosphere, the Ni-doped TiO_2_ nanotubes demonstrated a p-type semiconducting behavior, which was totally different from that of undoped TiO_2_ nanotubes. The Ni-doped nanotubes were found to be sensitive to a reducing atmosphere containing 1,000 ppm hydrogen at both room and relatively higher operating temperatures. The Ni-doped TiO_2_ nanotube sensor presented a good response reversibility and repeatability, as well as quick response after exposure to the hydrogen-containing atmosphere. Such a stable detection ability at low operating temperatures indicated that the microstructural modification with Ni-doping and nanotubular structures could facilitate the application of TiO_2_ based oxide in detection of hydrogen gas at relatively lower operating temperatures than previously reported.
